# The Beneficial Effects of Dietary Interventions on Gut Microbiota—An Up-to-Date Critical Review and Future Perspectives

**DOI:** 10.3390/nu15235005

**Published:** 2023-12-03

**Authors:** Carmen Purdel, Denisa Margină, Ines Adam-Dima, Anca Ungurianu

**Affiliations:** 1 Department of Toxicology, Faculty of Pharmacy, “Carol Davila” University of Medicine and Pharmacy, Traian Vuia 6, 020956 Bucharest, Romania; carmen.purdel@umfcd.ro (C.P.); elena.dima@umfcd.ro (I.A.-D.); 2 Department of Biochemistry, Faculty of Pharmacy, “Carol Davila” University of Medicine and Pharmacy, Traian Vuia 6, 020956 Bucharest, Romania; anca.ungurianu@umfcd.ro

**Keywords:** dietary intervention, fasting, caloric restriction, gut microbiota, interaction

## Abstract

Different dietary interventions, especially intermittent fasting, are widely used and promoted by physicians; these regimens have been studied lately for their impact on the gut microbiota composition/function and, consequently, on the general physiopathological processes of the host. Studies are showing that dietary components modulate the microbiota, and, at the same time, the host metabolism is deeply influenced by the different products resulting from nutrient transformation in the microbiota compartment. This reciprocal relationship can potentially influence even drug metabolism for chronic drug regimens, significantly impacting human health/disease. Recently, the influence of various dietary restrictions on the gut microbiota and the differences between the effects were investigated. In this review, we explored the current knowledge of different dietary restrictions on animal and human gut microbiota and the impact of these changes on human health.

## 1. Introduction

The human body is home to a great number of microbial cells found in diverse locations; their highest density is in the intestinal compartment, where they generate a complex microbial population known as the gut microbiota. This microbial community starts to develop after birth, evolves, and becomes more and more complex through infancy to eventually reach its adult form [1,2,3,4,5,6]. Its diversity is subject to different environmental factors, such as pH, oxygen levels/redox state, dietary nutrients, water activity, and temperature [5,6]. At birth, the gut microbiota is mainly composed of Proteobacteria and Actinobacteria; depending on the type of milk and then on food and lifestyle, it evolves through a stage where *Enterobacteriaceae*, *Bacteroides*, and *Bifidobacterium* are present. These are, in time, replaced mainly by members of the *Lachnospiraceae* and *Ruminococcaceae* families, and, in adults, the intestinal colonies are dominated by anaerobic bacteria from the major phyla of Firmicutes (predominantly *Lachnospiraceae and Ruminococcaceae*), Bacteroidetes, Actinobacteria, Proteobacteria, and Verrucomicrobia (*Akkermansia*) [3,5,6,7,8,9,10,11,12,13,14,15]. Furthermore, adults develop a personalised microbiota. Therefore, comparing microbiota composition between communities is challenging unless one population contrasts with different food cultures [16,17].

The gut microbiota is clearly involved in digestion, the fermentation of dietary fibers, and the production of some vitamins. In recent years, literature data have also shown that it is a central regulator of the host’s nutritional and metabolic homeostasis, influencing immunological functions. Dysbiosis is correlated not only with digestive issues or irritable bowel syndrome but is also involved in the etiopathology of obesity, type 2 diabetes mellitus (T2DM), and other metabolic imbalances, with a high incidence in the general population [18,19,20,21,22]. Interestingly, gut microbiota impairment is also associated with cardiovascular disease, neurodegeneration, nonalcoholic fatty liver disease, colon cancer, etc. For example, *Odoribacter* is negatively correlated with blood pressure [7,23], while *Escherichia coli* abundance is associated with endothelial dysfunction and metabolic syndrome [24,25,26].

Short-chain fatty acids (SCFAs) generated from the microbiota’s metabolism of nutrients are involved in numerous biochemical processes in the host [27]. Non-digestible carbohydrates (fibers) can be used in the colon as energy sources for local bacteria, who transform them into SCFAs (e.g., acetate, butyrate, and propionate), recognised for their involvement in different physiologic processes such as modulation of energy homeostasis, glucose/lipid metabolism, inflammation, and immunity [28,29,30]. Studies show that *Akkermansia municiphilla* species are important propionate-producing mucin-degrading organisms, while butyrate production is mainly attributed to the fermentation of resistant starches under the influence of *Ruminococcus bromii* but also to the metabolism of *Faecalibacterium prausnitzii*, *Eubacterium rectale*, and *Eubacterium hallii*; on the other hand, acetate production is not specific to certain species, being a pathway widely distributed among several bacterial groups [29,31,32,33,34].

Interestingly, the literature shows that SCFAs produced by microbial fermentation induce the same type of effects as increased plasma glucagon-like peptide-1 (GLP-1) and peptide YY (PYY) levels and also stimulate the secretion of gut peptides by acting on specific G-protein-coupled receptors expressed in cells abundant in the colon and terminal ileum [35,36,37,38,39,40,41].

Dietary interventions, as well as therapies aiming to correct microbiota imbalances, improve the metabolic function of humans [18,42]. Preclinical studies show that animals fed a diet rich in saturated lipids (lard) are characterised by a lower abundance and diversity of beneficial bacteria (*Akkermansia muciniphila*, *Lactobacillus*, and *Bifidobacterium*) compared to animals fed an isocaloric high-fat diet containing fish oil, rich in n-3 PUFA; this is associated with an increase in insulin resistance, inflammation, and consequent metabolic impairment [43]. These reports show that the interaction between the nutrients and the host microbiota is complex, with both components influencing each other. The microbiota can be altered by diet, but also the host metabolism can be modulated by the different metabolism products resulted from the microbiome transformation of nutrients since gut microbiota can perform many processes that cannot be supported by the host [44]. 

Knowledge about the repercussions of dietary changes on the animal and human gut microbiota is still insufficient. Furthermore, it is still unclear if the differences between animal models and humans regarding gut microbiota composition and dynamics limit the use of preclinical results. Although in clinical practise caloric restriction regimens are known to provide efficacy in reducing bodyweight and adiposity, reducing the risk of non-communicable comorbid disease [45,46], it is unclear if the results on gut microbiota composition observed after these regimens are more robust compared to time-restricted fasting. It is also unknown if the effects observed on gut microbiota composition are only temporary and how they impact the renewal of the intestinal mucosa. Furthermore, only a few studies have explored the interaction between the immune system and gut microbiota under different fasting conditions [47].

The present paper aims to review the recent literature data concerning the ability of dietary interventions currently used/recommended by the international medical community to influence/alter the taxonomic composition of gut microbiota and identify gaps in knowledge and potential avenues for a targeted, personalised diet.

## 2. Updated Concept of Dietary Intervention and Gut Microbiome Links

Various dietary caloric and/or time restriction regimens have been studied for their potential health benefits in healthy subjects, and in recent years, studies have also been performed in patients with different pathologies. In this narrative review, the term dietary intervention is used as an umbrella term for several strategies for restricting the time period of food consumption or the total caloric intake. The most common types of dietary interventions include time-restricted fasting (TRF), such as intermittent fasting (IF) or Ramadan fasting, and caloric restriction (CR) programs, such as alternate-day fasting (ADF), Buchinger fasting programs, water-only fasting, and fasting-mimicking diets (FMD) [48,49,50].

IF involves a fasting pattern at all times. The most popular versions of IF include (1) time-restricted fasting (e.g., the 16:8 method, which includes fasting for 16 h a day and having all meals within an 8 h window, or Ramadan fasting); (2) eat-stop-eat, which involves a 24 h fast once or twice per week; or (3) weekly fasting, like the 5:2 method, which involves unrestricted eating for five days of the week and restricting calories (usually to 500–600 kcal/day) for two non-consecutive days [51]. Alternate-day fasting involves alternating between days of no food restriction and days where food intake is severely restricted (<500 kcal/day) [52]. Many religious traditions, such as Ramadan in Islam (sunrise to sunset fasting) or Yom Kippur in Judaism, incorporate periods of fasting and are also considered IF [23].

There are several forms of CR where the energy intake is reduced to a fraction of the regular ad libitum intake or even to only water fasting. A particular CR is the Buchinger fasting protocol, which involves a primary fasting phase (200–300 kcal/day) that can last for several days (generally 5–7 days) to several weeks, depending on the individual’s health status and the specific goals of the fasting program. After this phase, participants gradually reintroduce solid foods into their diet (the transition phase). Furthermore, a new CR approach, the FMD, involves a low-calorie, low-protein, and high-fat diet to provide the beneficial effects of fasting while still allowing food intake [53,54,55].

In the last decades, studies on the impact of restriction programmes on several markers of metabolic health have been conducted; some of these studies also identified changes in gut microbiota composition. The potential effects of dietary restrictions on the gut microbiota could include shifts in microbial diversity (changes in the abundance and type of microbes) and metabolic alterations. Fasting or caloric restriction may alter the gut environment by changing the nutrient availability and energy sources, which can impact the growth of certain microbial phylum and influence SCFAs production.

**Figure 1:**
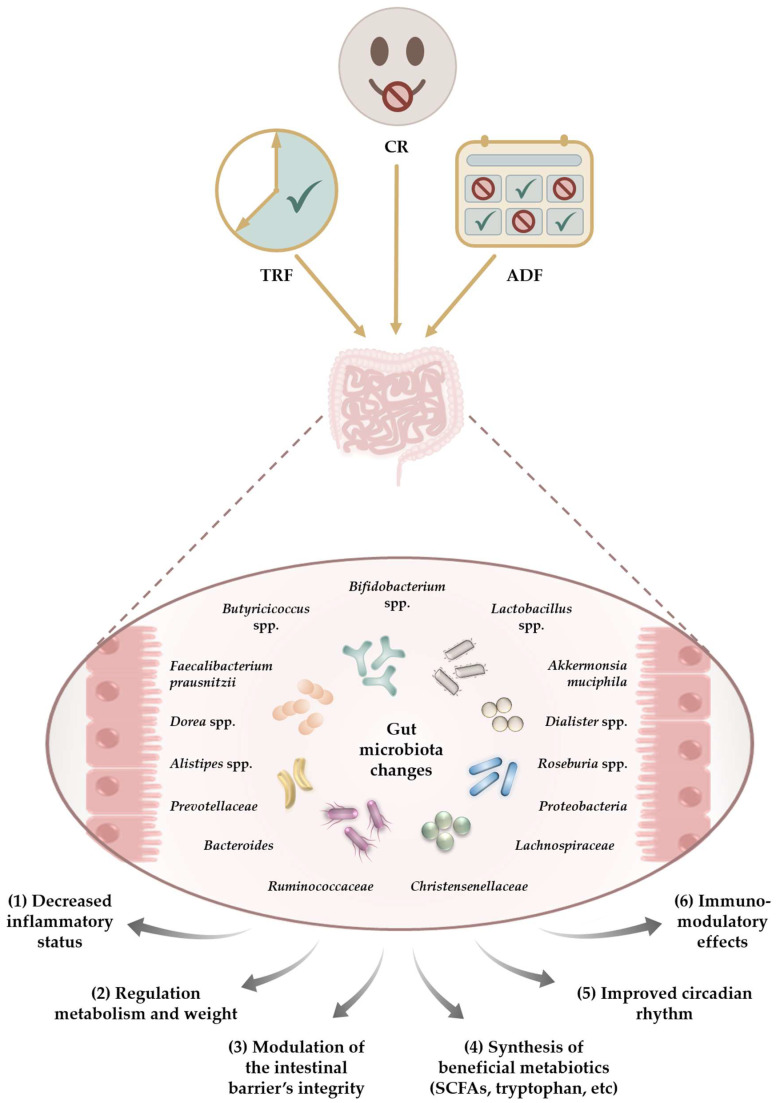
Potential beneficial effects of dietary interventions on the gut as the results of microbiota changes include TRF—time-restricted fasting; CR—caloric restriction; and ADF—alternate-day fasting.

The main health-beneficial effects as a result of microbiota changes are illustrated in Figure 1.

## 3. Methodology

For this narrative review, a literature survey was performed in PubMed to find the most relevant articles reporting interactions between dietary caloric restriction and gut microbiota. Articles were limited to those published in English, focusing on the most recent works between 2015 and 2023 (80.30% of the cited material). Additionally, the literature was reviewed to ascertain the key aspects regarding gut microbiota characteristics. For preclinical data, the publishing year was narrowed to a 2021–2023 interval, and the keywords and MeSH terms used were “gut microbiota” AND “fasted” AND “caloric restriction” AND “mice”, “rats” OR "animals." For clinical data, the applied search terms were “gut microbiota” AND “fasting” OR “intermittent fasting” OR “Ramadan fasting” OR “dietary caloric restriction” AND “clinical trial”.

Two researchers independently screened the titles and abstracts first, and disagreements were solved by discussion and consensus. Protocols, case reports, and studies where the main text was unavailable were excluded. Then, the full text of this papers was reviewed to retrieve the relevant information, and the most relevant papers were selected. The following information about each study was recorded: name of the first author, year of publication, study design, tested sample and analysing method, and main findings. A total of 17 papers regarding nonclinical data and 26 papers on clinical studies were selected after eligibility analysis, cross-checking, and removing duplicates.

In order to evaluate the gut microbiota alterations in various dietary conditions, the clinical studies were classified into two main groups: (1) Time-restricted fasting, including intermittent fasting or Ramadan fasting; and (2) Caloric restriction programs, such as alternate-day fasting, the Buchinger fasting program, water-only fasting, or a very low-calorie diet.

## 4. Effects of Dietary Intervention on the Animal Gut Microbiota

The repercussions of dietary intervention on the animal gut microbiota are still insufficiently known, although numerous published studies exist. As some excellent reviews have already been published [24], we strived to update and correlate the state-of-the-art data. The most recent research, published between 2021 and 2023, that investigated the alteration of gut microbiota in different animal models is summarised in Table 1.

Most fasting research is conducted using different animal models, and some interesting studies also involve hibernating animals. Song et al. investigated the gut microbiota of hibernating asiatic toads and observed temporal remodelling in the small and large intestines with discrepant patterns. For instance, Proteobacteria assembled in a higher proportion in the small intestine, while Firmicutes and Bacteroidetes assembled in a higher percentage in the large intestine. Also, the large intestine microbiota exhibited higher diversity than the small intestine one [60].

### 4.1. Studies Conducted on Healthy Animals

The microbial community in trout intestines can be significantly influenced by two weeks of feed deprivation [59]. Dysbiosis was characterised by a significant increase in Bacteroidetes and Firmicutes and a reduction in the Actinobacteria population. The effect of the feed restriction on relative diversity was more evident when considering family and genera than when considering phylum levels. The feed deprivation allowed the development of different commensal or pathogenic bacteria, such as Helicobacter species, responsible for several animal diseases [73]. Resuming normal feeding reestablished the microbiota balance and restored digestive functions.

Li et al. observed that gut microbiota composition changed when healthy mice were given a high-fat or CR diet (60% calories of the control group) and that the *Parabacteroides distasonis* population was significantly reduced by both diets. The CR diet induced a modified bile acid serum profile with a low fraction of non-12α-hydroxylated bile acids, ursodeoxycholic and lithocholic, which is linked to *P. distasonis* low abundance [56].

According to Zhang et al., which investigated fasting and refeeding in mice, *Akkermansia*, *Parabacteroides*, and *Muribaculaceae* abundance increased during fasting, whereas *Lactobacillus* and *Bifidobacterium* populations decreased. All the microbiota variations that occurred during fasting were reversible when refeeding ensued. *Akkermansia* was negatively correlated with plasma unconjugated primary and secondary bile acids and glucose levels but positively correlated with plasma conjugated secondary bile acids and faecal unconjugated forms of both bile acids. In contrast, *Lactobacillus* and *Bifidobacterium* were positively correlated with plasma levels of unconjugated primary bile acids and unconjugated secondary bile acids [57]. These results are supported by other authors who demonstrated the involvement of *Lactobacillus* and *Bifidobacterium* in bile acid metabolism, including deconjugation, via bile salt hydrolases or 7α-dehydroxylation [74]. 

Another study on rats revealed that TRF programs, such as once-a-day feeding for 28 weeks, significantly lowered the alpha diversity of the microbiota, which is an index of the overall community heterogenicity, reduced Actinobacteria and *Patescibacteria* abundance, and increased Verrucomicrobia abundance. TRF induced these qualitative and populational microbiota variations in young and old animals, irrespective of the feeding diet used (ketogenetic or regular) [58].

### 4.2. Studies Conducted on Different Animal Models

The protective effects of preoperative fasting were observed in a mouse model of intestinal ischemia and reperfusion that caused an increased abundance of *Helicobacteraceae* and *Ruminococcaceae* and decreased populations of *Muribaculaceae* and *Bacteroidaceae*. The 24 h fasting helped preserve the microbiota diversity, which was reduced by the intestinal injury, significantly increasing the abundance of Akkermansia, Ruminococcaceae-UCG-014 and Parabacteroides and decreasing the *Helicobacter* population, while a 6 h fasting may not be effective in changing the microbiota [67].

An IF (18:6) 5-week study on healthy rats increased alpha diversity while the Firmicutes/Bacteroidetes ratio was significantly diminished. From a qualitative point of view, the proportion of Proteobacteria was halved in the IF group versus the control group, while the *Lachnospiraceae* and *Lactobacillaceae* abundances were significantly higher [68]. 

In a mouse model of induced colitis, two IF programmes (short-term and long-term) determined different changes in the gut microbiota composition. Short-term IF increased the proportion of *Muribaculum*, *Bacteroides,* and *Akkermansia* and decreased the abundance of *Ruminiclostridium*, while long-term fasting decreased the abundance of *Akkermansia* and increased the abundance of *Lactobacillus*. Significantly, long-term IF reduced the severity of the colitis inflammation via SCFAs production. Additionally, the increased levels of inosine and secondary bile acids produced by *Akkermansia* are likely to contribute to the anti-colitis effects of IF [65].

Zhang et al. observed that the systemic inflammation in streptozotocin (STZ)-induced T2DM model rats was reduced by the CR program, as proven by decreased IL-6 and TNF-α levels. CR also modulated gut microbiota, increasing *Alistipes*, *Allobacum*, and *Lachnospiraceae* abundance, decreasing *Bacteroides*, *Lachnoclostridium*, and *Bifidobacterium*, and ameliorating hyperglycemia, possibly due to modulation of the overall structure of gut microbiota [66].

There are published data on obese animals, especially mice. When obese mice were subjected to the TRF programme for 12 weeks, significant variation of the faeces microbiota was noticed, as was the increased presence of Lactobacillus and Verrucomicrobiaceae represented by A. muciniphila, together with the reduction of lipid absorption related to the inhibition of the phosphoinositide 3-kinases/protein kinase B (PI3K/AKT) signaling pathway [69]. The increase in Lactobacillus abundance is associated with beneficial effects through multiple mechanisms, such as lactate synthesis or bile salt-hydrolase activity [75], while Akkermansia abundance is negatively correlated with insulin resistance and obesity [76].

In another study, a TRF regimen (16:8) for 16 weeks was found to be effective in reducing the negative effects of an obesogenic Western diet, such as hyperlipidemia, by preventing excessive hepatic lipid droplet accumulation and fibrosis, and also by reinstating cyclical fluctuations of gut genera such as *Lactobacillus*, *Faecalibacterium*, *Clostridia_UCG-014*, and by reducing *Helicobacter* abundance. Additionally, TRF helped restore the gut microbiota’s natural diurnal rhythms under different feeding conditions [63].

IF influences microbiome metabolism. In a rat model with pulmonary arterial hypertension, microbiome metabolites related to right ventricle mitochondrial dysfunction, such as bile acids and aromatic amino acid metabolites, were increased in the non-IF groups, unlike the IF group, where a normalisation in their concentrations was observed after 24 days of fasting. Furthermore, IF increased the abundance of *Lactobacillus* and balanced microbiome metabolite levels, suggesting that IF reversed the implications of the disease-induced alteration of the microbiome [70].

Zhang et al. compared, in C57BL/6 male mice, the effects of CR and IF with different dietary regimens on metabolic health and gut microbiota composition. The microbiota’s qualitative and quantitative composition varied with fasting and a rapid response. CR created a stable and unique gut microbial community, while IF resulted in dynamic gut microbiota changes during fasting-refeeding cycles. The IF regimen (5:2) with normal amounts of food on the feeding days positively affected glucose and lipid metabolism, similar to the CR regimen. The abundance of *Lactobacillus murinus* OTU2 increased regardless of dietary regimens [61]. *Lactobacillus murinus* abundance is correlated with multiple metabolic improvements and decreased levels of inflammatory markers [77,78].

When assessing the effects of different IF programmes on the gut microbiota in a mouse animal model of allergy, *Alistipes* and *Odoribacter* were the most abundant genera among all the species. However, their populations increased differently: *Alistipes* in the IF (24:24) group and *Odoribacter* in the IF (16:8) group. The IF (24:24) regimen reshaped the gut microbiota, with a higher abundance of *Alistipes* and *Rikenellaceae* strains than the other regimen. Both IF regimens can reduce the production of IgE and IgG1, a type of antibody that plays a crucial role in allergic reactions [62]. Furthermore, *Odoribacter* contributes to the increased production of SCFAs and exhibits a protective anti-alergic effect [79,80]. These results can create premises for similar research in humans with allergies, although an IF (24:24) regimen might be challenging to implement.

Nowadays, it is well established that the gut microbiota is linked to brain functioning, and the gut microbiome is a component of the gut-brain axis. Communication between the gut microbiota and the central nervous system can occur through several signalling molecules, including SCFAs, folate, and tryptophan metabolites [81,82].

Changes in the gut microbiota may affect cognitive function, but the exact mechanisms are not fully understood. 

Some authors tried to investigate whether longer TRF influences gut microbiome diversity and enhances cognitive function in young and old rats. In a 13-month TRF regimen, rat microbiota diversity significantly changed, with a particular decrease in the *Allobaculum* population, together with better cognitive performance. TRF had the most significant influence on cognitive performance in aged rats compared to young animals [83]. As *Allobaculum* is a SCFA producer, including butyric acid [84,85], the correlation between its abundance and increased cognitive task performance is plausible. In humans, there is evidence that manipulating the gut microbiota may enhance cognitive flexibility and executive function [86].

Furthermore, whether combined or separately, IF and aerobic exercise can impact the gut-brain axis. Specifically, Soare et al. evaluated rats that underwent IF or “trained” IF (IF and aerobic exercise) for four weeks and observed improvements in somatic and behavioural anxiety and depression parameters. Trained IF demonstrated a tendency towards an anxiolytic effect, while sedentary IF showed a potential antidepressant effect. Also, the trained IF group noticed lower counts of beneficial bacteria such as *Bifidobacterium* and *Lactobacillus*, while sedentary IF showed higher *Bifidobacterium* and *Enterococcus* counts [71].

Liang et al. observed that maternal long-term IF in mice for 12 weeks has negative metabolic consequences for the offspring in adulthood, mainly due to the marked reduction of the *Lactobacillus intestinalis* population. The reduction of L. *intestinalis* suppressed the expression of the intestinal tight junction protein Claudin-1, disrupted intestinal barrier integrity, and generated metabolic disturbances such as impaired glucose tolerance, adiposity, and advanced hepatic steatosis [64].

Interestingly, Graef et al. observed that fasting led to changes in the gut microbiota, which were associated with a reduction in *Salmonella* pathogen load, specifically in the cecum, but not in the stomach or small intestine of fasted mice. The fasting altered the gut microbiota by increasing the *Akkermansia population and reducing* the relative abundance of *Bacilli* and *Erysipelotrichia*. Fasting was found to protect hosts from intestinal bacterial infections, in part through the actions of the gut microbiome [72].

The main findings on the animal models are illustrated in Table 2.

Comparing the information regarding the variation of different types of bacteria in animals, the most frequently studied phylum was Firmicutes. However, the results are contradictory, as some studies reported an increase in the abundance of Firmicutes [56,59,60,71], but others mentioned the reduction of its proportion in the gut microbiota after 24:24 h or 16:8 h IF regimens [62,65,67]. Another phylum of great interest is Bacteroidetes, which is often assessed in conjunction with Firmicutes. Among the studies that refer to the Bacteroidetes levels, three have observed a corresponding increase [59,60,65,71], whereas one study also indicated a decrease in Firmicutes [65]. The decreased Firmicutes/Bacteroidetes ratio linked to IF was previously identified by other researchers [68,87].

*The Lactobacillus* genus, which is considered highly relevant for the microbiome composition and is generally linked to improved health status, was increased by TRF in five reports [63,65,68,69,70], while in the other three, it was lowered [57,64,71]. The results obtained for the *Akkermansia* population are also heterogeneous. IF resulted in increased *Akkermansia* abundance in five studies, mainly comprising short-term IF [57,65,67,69,72], but at lower levels during long-term IF [65]. Similar inconsistencies are observed in studies that reported variations of other genera, such as *Clostridium* or *Allobaculum* [58,66].

As a result, the results of animal studies indicate that gut microbiota composition is adaptive to feeding regimen modifications. Nevertheless, the results seem to lack homogeneity and coherence regarding individual bacteria strain/group dynamics. Moreover, some data suggests that different fasting regimens exhibit different effects on the microbiota, and not always long-term changes. Drawing specific conclusions about the consequences stemming from particular feeding regimens is challenging, given that the experimental protocols encompass a variety of feeding restrictions, different durations, and diverse diets. Additionally, examinations were conducted on various animal models, which may exhibit specific sensitivity to CR. The microbiota was analysed using faecal samples obtained from different digestive tract segments, with varying properties in different animal species. Therefore, interpretation of the preclinical results should be made with caution and in conjunction with human data.

## 5. Effect of Dietary Interventions on the Human Gut Microbiota

The impact of different restriction regimens on the gut microbiota was investigated in healthy volunteers as well as in overweight, hypertensive, or metabolic syndrome patients. A total of 11 studies were identified in healthy volunteers, of which four evaluated the effects of CR regimens and seven investigated the influence of TRF, mainly Ramadan IF. In patients, 10 studies were identified, of which 6 evaluated the effects of CR and 4 investigated the influence of TRF regimens. The main findings are summarised in Table 3.

### 5.1. Studies Conducted on Healthy Volunteers

#### 5.1.1. CR Regimens

In a randomised, controlled, single-blinded trial, Lilja et al. compared the gut microbiome before and after a five-day Buchinger fasting programme in healthy individuals with three months of supplementation containing sirtuin (SIRT)-activating nutraceuticals (SIRTFOOD intervention), SIRTs being at the forefront of energy metabolism, autophagy, and senescence [107,108]. Gut composition and metabolites were investigated using Illumnia sequencing and mass spectrometry of stool samples. In the Buchinger fasting group, Proteobacteria distribution increased while the Firmicutes/Bacteroidetes ratio decreased and was correlated with the body mass index (BMI) change. No changes for Actinobacteria were seen after the fasting intervention, but after the SIRTFOOD intervention, a different pattern was observed as Actinobacteria increased [88].

Later, the same authors observed that the Buchinger fasting programme also affects SIRT expression in healthy humans. Following fasting, the *Firmicutes*/Bacteroidetes ratio decreased. The participants with a higher abundance of *Prevotella* or *Lactobacillus* had higher levels of *SIRT1* expression at baseline, but the correlation was not observed after the intervention. The abundance of *Christensenellaceae* species, which are butyrate producers, increased after fasting and was positively correlated with *SIRT3* expression [89]. SIRT1 and SIRT3 are longevity-related proteins linked with mitochondrial biogenesis and mitophagy for damaged mitochondria [109,110]; therefore, fasting may have beneficial outcomes for human health and influence some longevity-associated mechanisms.

Mesnage et al. investigated the effects of a ten-day Buchinger fast on the gut microbiota of fifteen healthy volunteers and the subsequent 3-month refeeding effects. The stool was assessed with 16S rRNA gene amplicon sequencing. There were no differences in alpha diversity. Fasting caused an initial decrease in the abundance of Firmicutes, such as *Lachnospiraceae* and *Ruminococcaceae*, bacteria known to degrade dietary polysaccharides. There was a concomitant increase in Bacteroides and Proteobacteria abundance (*Escherichia coli* and *Bilophila wadsworthia*). The inversion of the Firmicutes/Bacteroidetes ratio was due to a significant decrease in the relative abundance of Firmicutes. The gut microbiota changes were reversed after three months. The levels of faecal branched-chain amino acids (BCAA) significantly increased during fasting, returned to baseline after refeeding, and declined significantly after three months [91]. The association between Bacteroidetes abundance and faecal BCAA levels suggests that using host-derived compounds, such as desquamated cells, could sustain energy requirements during fasting.

He et al. investigated the effects of two fasting models, water-only and juice-fast, for seven days in 16 healthy individuals. The stools were assessed using 16S rRNA gene sequencing. The water-only fasting dramatically changed the bacterial community, and participants developed a more homogenous gut microbiome and reduced the relative abundance of Fusobacterium. Furthermore, post-fasting *Fusobacterium* remained consistently low in all water-only fasting participants. Although the authors anticipated an increase in the relative abundance of *Akkermansia* in the water-only fast group, this was not observed since *Akkermansia* uses mucin as its sole substrate. The lack of this effect suggests that other bacteria could utilise mucin, which may compete with *Akkermansia* [90].

#### 5.1.2. TRF Regimens

In a cohort trial, Su et al. evaluated the effect of the 30-day Ramadan fasting on the gut microbiota in healthy young and middle-aged individuals. Fasting led to substantial remodelling of the gut microbiota in both cohorts, supporting increased alpha diversity only in the younger group. Increased microbiome diversity was specifically associated with a reversible upregulation of the Clostridiales order-derived *Lachnospiraceae* and *Ruminococcaceae* families, while the abundance of the Prevotellaceae family decreased. As Lachnospiraceae species are linked to intestinal butyrate production, their increase positively affects blood glucose, body weight, and body fat mass. Microbiome composition returned to baseline during the follow-up phase [7].

Ozkul et al. investigated the microbiota alterations in nine volunteers undergoing Ramadan IF using 16S sequencing. Microbial richness increased, and elevated levels of SCFA producers, including *Faecalibacterium prausnitzii*, *Roseburia*, *Eubacterium*, and *Akkermansia*, were observed after the fasting. The most affected was *Butyricicoccus pullicaecorum*, one of the main butyrate-producing bacterial species. Although the Firmicutes/Bacteroidetes ratio remained increased, a higher abundance of Bacteroidetes was noticed compared to baseline [92]. These findings are in line with another similar Ramadan-based study published by the same researchers that reported an increased abundance of *A. muciniphila* and the *Bacteroides fragilis* group [23]. Furthermore, fasting significantly reduced fasting serum glucose and total cholesterol levels in all volunteers.

Ali et al. investigated microbiota changes throughout Ramadan IF in two cohorts of Chinese and Pakistani individuals using 16S sequencing of stool samples [93]. No effect on alpha diversity was observed, and other fast-related changes differ substantially between the subcohorts. Specifically, *Prevotella* and *Faecalibacterium* drove the predominance of Bacteroidetes and Firmicutes in the Pakistani group, while *Bacteroides* were the most prevalent among Chinese individuals. *Dorea*, *Klebsiella*, and *Faecalibacterium* were abundant in the Chinese group after fasting, while *Sutterella*, *Parabacteroides*, and *Alistipes* were significantly enriched after fasting in the Pakistani group. The prevalence of *Sutterella* suggests a potential metabolic benefit based on the positive impacts on glucose levels, while *Parabacteroides* are considered a potential factor associated with the inhibition of weight gain. Also, the genera *Coprococcus*, *Clostridium_XlV*, and *Lachnospiracea* significantly decreased after fasting. Within the Chinese individuals, *Faecalibacterium* increased after fasting, which is comparable with the findings of Ozkul et al. [92].

Although Mindikoglu et al. found no differences in bacterial richness and alpha diversity throughout IF Ramadan in fourteen healthy subjects, serum proteomic profiling showed that fasting upregulated the gene-protein products associated with glucose and lipid metabolism and insulin signaling. The upregulation of PKM M1/2 (pyruvate kinase M1/2, an enzyme involved in glycolysis) and PLIN4 (perilipin 4, a protein linked with cell lipid storage) expression indicates that IF may be highly effective in managing metabolic syndrome. Furthermore, NR1D1 (nuclear receptor subfamily 1 group D member 1) and ASGR2 GP (asialoglycoprotein receptor) levels increased significantly after the Ramadan fasting period, which may suggest that IF can also have a protective role in hepatic steatosis [111].

In a comparative cross-sectional study, Mohammadzadeh et al. investigated the consequences of Ramadan IF in thirty healthy subjects on gut microbiota and serum butyrate concentration. The study showed that gut *Bacteroides* and Firmicutes increased by 21% and 13% after fasting compared to baseline. The increment in *Bacteroides* occurred in both sexes, but Firmicutes significantly increased only in women. Also, the serum levels of butyrate significantly increased after IF. Furthermore, the BMI decrease after Ramadan fasting was associated with an increased abundance of Bacteroides in the gut microbiota [94]. *In a cohort study on two ethnic groups* (Chinese and Pakistani), Chen et al. *observed shifts in faecal metabolite profiles after Ramadan fasting*. Some metabolite levels (L-histidine, cordycepin, and lycofawcine) were significantly higher in the Chinese cohort, while brucine increased in the Pakistani group after IF. Several bacterial taxa were significantly correlated with specific metabolites unique to each ethnic group. The authors suggested that the observed changes in faecal metabolite profiles could be influenced by associated shifts in the gut microbiota [95].

Zeb et al. divided thirty healthy men into TRF and a control group (non-TRF) and investigated the effects of TRF (16:8) over 12 weeks on the gut microbiota. TRF significantly changed microbial diversity, increasing the Bacteroidetes phylum (*Prevotella_9*, *Faecalibacterium*, and *Dialister*) compared to the control. In the TRF group, Bacteroidetes was the most abundant phylum, followed by Firmicutes; this ratio suggests the beneficial effect of TRF on gut flora [96]. The same researchers also found that enhanced gut microbiota richness, such as *Prevotellaceae*, is linked to the activation of SIRT1 [112].

### 5.2. Studies Conducted on Obese Patients

#### 5.2.1. CR Regimens

In a pilot study on 13 obese patients, Remely et al. [97] investigated the effect of a 1-week Buchinger fasting programme with laxative treatment followed by a 6-week intervention with an additional probiotic mixture of *Lactobacillus* sp., *Streptococus thermophiles*, and *Bifidobacterium* sp. Gut microbiota (the relative abundance or composition) were analysed based on 16s rDNA with a quantitative real-time polymerase chain reaction (qPCR). No significant changes in the abundance of Prevotella, Bacteroidetes, Clostridium cluster XIVa, or Clostridium cluster IV were found. However, SCFAs producing mucus-associated Faecalibacterium prausnitzii, Bifidobacteria, and Akkermanisa increased in abundance over this study period and influenced the integrity of the intestinal epithelial barrier. The inflammation-associated gut microbes *Lactobacilli* and *Enterobacteria* increased during the first week but then declined by the end of the intervention. The addition of probiotics increased gut microbial populations. The findings on *Faecalibacterium prausnitzii* and *Akkermansia* are comparable with Ozkul et al.’s results in healthy volunteers [92].

Maifeld et al. investigated, in a randomised controlled trial, the effect of a 5-day Buchinger fasting program, followed by a Mediterranean-like diet for three months, in hypertensive patients with metabolic syndrome. Fasting modified the gut microbiota, decreasing SCFA producers such as *Bifidobacterium*, *Coprococcus comes*, and *Roseburia*, increasing F. prausnitzii, *Bacteroides*, and *Anaerotruncus*, and also reducing propionate production capacity as well as mucin degradation gene modules. These changes had beneficial effects on body weight and blood pressure. No significant changes in alpha diversity were observed, and most of the microbiome alterations were reversed after three months of refeeding [98].

Guo et al. observed gut microbiota changes after eight weeks of IF (5:2) compared with the control group in a randomised clinical trial in 39 adult patients with metabolic syndrome. IF significantly increased the relative abundances of SCFA producers, such as *Ruminococcaceae*, *Roseburia*, and *Clostridium*, at the genus and family levels. Significant alterations of the gut microbiota were found at the species level. Intermittent fasting mainly induced a shift in Firmicutes but also decreased the circulating levels of lipopolysaccharides [99].

Alemán et al. assessed the gut microbiome composition through 16S rRNA sequencing of faecal microbiota in 10 obese postmenopausal women during a very low-calorie diet (800 kcal/day) for 46 days. An overall parallel shift in community structure was observed, corresponding to a reduced abundance of *Faecalibacterium prausnitzii* and the *Roseburia* genus and increased *Christensenellaceae*. Furthermore, changes in microbial taxa were correlated with faecal bile acid composition variations. A strong negative correlation between *Clostridiaceae* and lithocholic acid and positive correlations between *Eubacterium* and lithocholic acid were observed. Isolithocholic correlated negatively with *Clostridiaceae* and *Ruminococcus*, while *Faecalibacterium* showed strong positive correlations with murocholic acid and ursodeoxycholic acid [101]. The authors also observed that during this intervention, the increased lipolysis led to higher beta-hydroxybutyrate levels, an SCFA negatively correlated with *F. prausnitzii* and the genus *Roseburia. These findings contrast with the results of other studies*, but the reduced number of participants should be taken into account.

In a randomised trial of a group of obese patients, a 6-week VLCD (very low-calorie diet) resulted in an altered faecal microbiota, particularly *Bacteroides* spp., which decreased approximately two logs compared to baseline, and the *Lactobacillus* group with a reduction of approximately 1 log, but the change was transient. VLCD was also associated with a decrease in body weight. The normalisation of microbiota observed at the 12-month follow-up suggested that microbiota modifications were associated with dietary intake rather than with body weight variations [104]. Also, the effect of a long-term CR regimen was studied. Ruiz et al. observed that one year of CR impacts gut microbial composition in obese/overweight adolescents [105]. Long-term CR induced significant shifts in total and β-galactosidase-containing species, reduced Actinobacteria and the Firmicutes/Bacteroidetes ratio, and enhanced beneficial *Bacteroides*, *Roseburia*, *Faecalibacterium*, and *Clostridium* XIVa, all playing an essential functional role in the polysaccharide trophic chain. Moreover, the post-CR gut microbial community of obese adolescents exhibited similar metabolic performances compared with the lean control, suggesting a high level of metabolic plasticity [113].

#### 5.2.2. TRF Regimens

Khan et al. included fourteen women and thirty-one men in a TRF regimen (16:8) for 16 days. The volunteers were divided based on their weight into overweight/obese, normal, or underweight groups [100]. The human faecal bacterial diversity exhibited significant changes with increased alpha diversity. The number of *Bifidobacteria* and *Lactobacillus* increased in all groups. In the female overweight/obese group, Firmicutes, Bacteroidetes, and Actinobacteria decreased, while Proteobacteria increased. In contrast, in the normal or underweight female groups, Firmicutes, Proteobacteria, Bacteroidetes, and Actinobacteria exhibited the same pattern as in the obese group. The effect of intermittent fasting on male volunteers is unique for both groups. In the male normal group, Firmicutes and Actinobacteria decreased, while Proteobacteria and Bacteroidetes increased, while in the underweight group, Firmicutes, Bacteroidetes, and Actinobacteria increased, and Proteobacteria decreased in response to TRF. These results suggest that IFs impact could be gender-specific, but more in-depth studies are needed to confirm this hypothesis. Furthermore, the ameliorated blood lipid profile is positively correlated with increased *Lactobacillus* and *Bifidobacterium* spp., which exhibit bile salt hydrolases that influence cholesterol absorption [18].

Gabel et al. examined in a 12-week pilot study the changes in the gut microbiome in obese adults after IF [114]. Participants underwent a TRF (16:8) intervention, and the faecal microbiota was determined by 16S rRNA gene sequencing. At baseline, the two most common phyla were Bacteroidetes and Firmicutes at 26.9% and 61.2%, respectively. No significant alterations in the abundance of common phyla were observed after 12 weeks of TRF. These findings contradict other published data [103,115], which have all reported changes in gut microbiota composition and/or diversity after 6 or 12 weeks of caloric restriction. The authors concluded that the CR used in their study was insufficient to have a beneficial impact on gut microbiota composition.

Dao et al. evaluated the effect of a 6-week CR and a 6-week weight stabilisation diet on faecal *Akkermansia muciniphila* abundance and faecal microbial gene richness in 49 obese adults [102]. At baseline, there were two groups: one with a high *A. muciniphila* abundance (Akk HI group) and the other with a lower abundance (Akk LO group). The Akk HI group exhibited the healthiest metabolic status, assessed by fasting plasma glucose, plasma triglycerides, and body fat distribution. While in the Akk HI group, *A. muciniphila* abundance decreased after CR and the total intervention period but remained significantly higher compared with the Akk LO group (more than 100-fold).

Frost et al. investigated in a pilot study in obese type 2 diabetics the effect of a 6-week VLCD followed by a nine-week food reintroduction and stabilisation period. The tests were performed on the faecal microbiota using 16S rRNA gene sequencing. From the reported data, alpha diversity increased, along with the depletion of some pathobiont taxa, like *Collinsella*, which decreased 8.4-fold, *Roseburia*, and *Lachnospiraceae* spp. As the *Collinsella* genus has been associated with poor metabolic status and T2DM, the reduced abundance of *Collinsella* could merely represent a biomarker indicating an improved metabolic state. Most microbiome changes observed had reverted until the end of this study [103].

Also, Jian et al. observed that eight weeks of caloric restriction (800–1200 kcal/day) in obese patients with pre-diabetes induced significant changes in gut microbiota, including increased levels of *Akkermansia* and *Christensenellaceae* R-7 groups and decreased levels of *Blautia* and *Bifidobacterium* spp. The changes in microbiota composition were significantly associated with weight loss [106].

## 6. Comparability between Dietary Intervention Regimens

A few studies investigate if different dietary programmes may have a common pattern of gut microbiota changes. These are summarised in Table 4.

Stanislawski et al. investigated the gut microbiota changes in overweight subjects recruited for the DRIFT2 trial. DRIFT2 compares the weight loss achieved by IF to that by CR. Gut microbiome analysis through 16S sequencing was performed for the first three months. The overall microbiota community structure (beta diversity) shifted significantly from baseline to three months. *Collinsella* and *Subdoligranulum* decreased in relative abundance, while *Parabacteroides*, *Alistipes*, and *Bacteroides* increased. *Akkermansia* increased significantly among IF participants but showed no significant change in daily CR [116].

Gutiérrez-Repiso et al. included 61 individuals who followed three different weight loss strategies (bariatric surgery, the Mediterranean diet, and a ketogenic very-low-calorie ketogenic diet, VLCKD), and the gut microbiota profile was assessed by next-generation sequencing. While microbiome changes occurred in each type of intervention, a common taxon could not be found. At the family level, *Clostiridiaceae* significantly increased their abundance with the Mediterranean diet and VLCKD, while *Porphyromonadacean* and *Rikenellaceae* significantly increased their abundance with VLCKD and bariatric surgery. At the genus level, *Parabacteroides* and *Alistipes* significantly increased their abundance, while *Lactobacillus* decreased with a caloric restriction diet and bariatric surgery. Predicted metagenome analysis suggested that most changes after the CR diet focused on biosynthesis and degradation/utilisation pathways, while enrichment characterised the Mediterranean diet in several pathways related of the fermentation to SCFAs. Bariatric surgery seems to decrease most of the biosynthesis pathways as a sign of extreme caloric restriction [118]. The same authors revealed that VLCKD changes in the microbiota are more significant if probiotics (such as *Bifidobacterium lactis*) and prebiotic fibers are supplemented during the diet [121].

Recently, in a real-life study, Ferrocino et al. compared the effects of a 12-week TRF regimen (<12 h feeding) with a CR (500–1000 kcal/day) in 49 obese patients. No differences in alpha and beta diversity or gut microbiota composition were observed. *Lachnospiraceae*, *Parasutterella*, and *Romboutsia* frequencies significantly increased in the TRE group [117]. The upregulation of *Lachnospiraceae* has already been mentioned after Ramadan fasting [7] and interpreted as a possible explanation for the health benefits of TRE, considering their butyric-acid-producing capacity. 

Siebert et al. investigated the effect of continuous caloric restriction, or IF, on overweight or obese patients who participated in a 1-year behavioural weight loss intervention. Although the authors did not report between-group differences, they identified four gut microbes as predictors of change in weight: improved weight loss was correlated with *Ruminococcaeae* NK4A214 and *Coprococcus* 3 baseline abundance. At the same time, Bacteroides and *Lachnospiraceae* levels were considered disadvantageous for weight loss. *Faecalibacterium* and *Blautia* baseline abundances were associated with a decrease in triglicerides [119].

These findings regarding *Blautia* and *Faecalibacterium* dynamics and their beneficial effects can be seen in conjunction with another study on patients with other pathologies. Cignarella et al. initiated a 15-day randomised controlled pilot trial for multiple sclerosis subjects experiencing relapse; seventeen subjects were equally randomised to alternate-day fasting vs. an ad libitum diet [122]. In the stool samples, no bacteria were significantly different at day 15 between the two groups, but the abundance of *Lachnospiraceae*, *Blautia,* and *Faecalibacterium* showed an increasing trend in the IF group. As *Faecalibacterium* and *Blautia*, which belong to the Clostridia clusters XIV and XIVa, play an important role in producing butyrate in the gut, the increase of these genera might counterbalance the gut dysbiosis usually detected in multiple sclerosis [123].

Sowah et al. analysed the effects of intermittent CR (5:2) versus continuous CR on the faecal microbiota of 147 obese adults in a 50-week parallel-arm randomised controlled study. Except for *Lactobacillales* being enriched after intermittent CR, post-intervention microbiome composition did not significantly differ between groups. Despite the lack of effects on microbiota composition, the linear mixed effect analysis, which leveraged data across all time points and participants, revealed associations between HOMA-IR and *Akkermansiaceae*, *Christensenellaceae*, and *Tanerellaceae*, underscoring the potential importance of these bacteria for host metabolic status [120].

## 7. The Impact of CR Programs on the Gut Microbiota—Common Findings

The main findings of studies involving human subjects are illustrated in Table 5.

Comparing the above data, the most frequent change reported is an enrichment of *Faecalibacterium prausnitzii*, a member of Firmicutes, a well-known SCFA producer. The enrichment was observed in five studies [7,92,93,96,105,118], but also decreased in the other two trials [91,101]. *F. prausnitzii* exhibits beneficial effects by producing anti-inflammatory molecules such as shikimic and salicylic acids [108] and modulating the intestinal mucus barrier by modifying goblet cells and mucin glycosylation [124]. It is also positively correlated with obesity as it provides additional energy for gut epithelial cells and has a role in adipose tissue expansion [125]. Also, a low-fat fat/high-carbohydrate diet increased the abundance of *F. prausnitzii* in obese patients [126], but not in patients with metabolic syndrome [127], which suggests that the degree of dysbiosis associated with the disease may be a determinant factor in response to a specific diet-based treatment. Although changes in *F. prausnitzii* abundance were not consistent among the studies, there is sufficient evidence to use *F. prausnitzii* as an indicator or biomarker of human intestinal health.

Furthermore, other SCFA producers, such as *Roseburia*, decreased in four CR programs [91,101,103,118], and increased during refeeding in the other three studies [92,99,105]. Also, *Butyricicoccus pullicaecorum* abundance displayed a similar variable pattern, increasing during fasting [92,99]. As it is well known that *Roseburia*, *F. prausnitzii*, and *B. pullicaecorum* influence the integrity of the intestinal epithelial barrier and support immunity, these beneficial changes should be taken into account in further research.

The relative abundance of *Akkermansia muciniphila*, another SCFA producer and mucin-degrading bacterium, increased during different restriction programmes in five clinical trials [23,92,97,106,116]. As the level of *A. muciniphila* is inversely correlated with body weight and directly correlated with the weight loss observed in obese patients after CR restrictions and improvements in multiple indicators of cardio-metabolic health [128,129,130,131], it can be proposed as a prognostic tool in the context of predicting CR success. Further studies are needed to confirm this, as the effect was not consistently observed in all trials. The difference between restriction programs, especially the type and fibre content of the diet, can partially explain controversial results. The abundance of *Akkermansia muciniphila* increases in the absence of fibre polysaccharides [132], and this may explain Ramadan IF results since the Ramadan diet includes low fibre intake [23]. Recently, it was observed that, in addition to its metabolic and anti-inflammatory effects, *Akkermansia muciniphila* influences cognitive functions in patients with type 2 diabetes; therefore, increasing its abundance via fasting or the use of metformin [133] may be protective against cognitive decline in elderly type 2 diabetics.

Gut alpha diversity increased only in some studies after Ramadan IF or VLCD, but fasting in general did not influence it. As alpha diversity is considered a marker of gut health and homeostasis, the results indicate that dietary restrictions are not expected to have a negative impact on gut microbiota diversity.

Although an increase in *Alisipes* abundance was observed in a significant number of studies, considering that the role of *Alisipes* is not fully elucidated, these results have limited value. *Alisipes* exhibit protective effects against colitis or cardiovascular disease but are also considered to have a pathogenic role in colorectal cancer and are positively correlated with signs of anxiety or depression [134].

*Bacteroides* increased after TRF, including Ramadan IF or long-term CR, but decreased during refeeding. Furthermore, the *Bacteroides* abundance is linked to the Firmicutes/Bacteroides ratio, a marker of normal intestinal homeostasis. This ratio decreased in three trials after the Buchinger fasting programmes or long-term CR but did not change after VLCD. Interestingly, in one study, it remained increased after Ramadan IF [92].

Several studies reported a positive impact on butyrate-producing bacteria (such as *Ruminococcaceae* or *Lachnospiraceae*), while others showed no significant change in the relative abundances of such species. Therefore, a clear trend cannot be set, and further data are needed. 

Also, *Bifidobacterium* and *Lactobacillus* species increased in obese patients after the CR regimen [97,120] but also decreased [106], with inconsistent results. 

*Prevotella* abundance is also positively influenced by Ramadan IF, and considering it is linked to the activation of SIRT1, together with *Diallister* and Bacteroidia, it may influence and control circadian rhythmicity that regulates intestinal physiology [112,135].

The enrichment of the *Parabacteriodes* population observed in some studies should be mentioned because it is also linked with its capacity to conjugate bile acids and influence their biological functions, including controlling metabolic dysfunctions [136].

Although no clear trend or common pattern of gut microbiota changes can be established as a result of all different dietary approaches, some health-beneficial effects as a result of microbiota changes are apparent, including a reduction of risk factors for a range of age-related diseases and increased lifespan. Dietary restriction influences the gut microbiota by decreasing proinflammatory cytokines such as IFN-gamma and TNF-α [137] or enhancing SCFA production [108]. It also increases intestinal barrier integrity by stimulating mucin production and exhibits immunomodulatory effects by influencing the abundance of *Roseburia* and *F. prausnitzii* [138]. Furthermore, dietary restriction improves circadian rhythm via enhanced SCFA production or activation of SIRT1 [112,135]. It protects against metabolic illness via *Lactobacillus* and *Oscillibacter* spp., which regulate body weight and glucose and lipid metabolism by influencing the release of GLP-1 and PYY [139]. 

A beneficial and noteworthy effect induced by dietary changes—the counteracting of aging—was highlighted in some studies. The pro-longevity and regenerative effects of IF or CR could be partially connected to gut microbiota changes. In humans, ageing is linked with a decrease in *Bifidobacteria*, *Ruminococcaceae*, *Lachnospiraceae*, and *Bacteridaceae* [140,141]. Simultaneously, extremely long-lived individuals exhibit an increase in several beneficial taxa, particularly *Akkermansia*, *Bifidobacterium*, and *Christensenellaceae*, which are now considered a “signature of longevity” [142,143]. Therefore, the increased abundance of *Akkermansia muciniphila* observed after Ramadan IF or CR or the positive impact on *Ruminococcaceae* or *Lachnospiraceae* observed in some studies supports this hypothesis. In addition, the pro-longevity effects induced by IF or CR are also linked with other mechanisms, such as reducing circulating insulin-like growth factor-1 (IGF-1) levels, protein-kinase A activity, or mTOR pathways [144,145,146].

The heterogeneity of the human results is correlated with the fact that the studies were conducted either in healthy volunteers or in overweight patients. The microbiota of an overweight patient is significantly different from that of a normal-weight individual and characterised by a lower alpha diversity and increased abundance of Actinobacteria, Bacteroidetes, and Firmicutes phylum species [147,148,149]. This different status explains, for example, why, only in obese patients, Actinobacteria decreased in response to IF.

The impact of dietary caloric restrictions in the context of everyday life is still under debate, as many dietary restrictions are indicated by physicians in patients with different pathologies (obesity, hypertension, T2DM, cancer), which are also under specific medication, and gut microbiota can, directly and indirectly, affect drug metabolism [150]. Furthermore, drugs may directly alter the abundance of specific bacterial species [151]. This bidirectional interaction should be further investigated, especially regarding the commonly used drugs and their impact on their efficacy.

## 8. Conclusions

Considering that in humans, fasting and/or caloric restriction are often exercised on a daily basis for different reasons, and there is a significant interest worldwide in its influence on general health, we reviewed the recent literature data concerning the ability of different dietary interventions to influence the taxonomic composition of the gut microbiota. Although the research in the field is not abundant, most studies suggest beneficial effects, but the available data are insufficient to establish a typical pattern of gut microbiota changes induced by different dietary approaches. Furthermore, it is impossible to conclude if a certain type of restriction showed more consistent beneficial results on the gut microbiota than the others. This was expected due to the heterogeneity and low power of the studies, microbiota baseline variability, and varying individual responses. Furthermore, many published studies do not consider common confounders, such as smoking or physical exercise. Only a few studies used a control group or investigated if the beneficial effects may be reversible and require continued restricted eating behaviour. In addition, these beneficial effects should be carefully weighed against the risks induced by fasting, especially by Ramadan IF, which perturbs the human circadian rhythms and may negatively influence human health in the long run. Further research in this area is necessary, particularly on obese and metabolically compromised patients, as they show a low fluctuation of circadian rhythm. Also, the risk of developing an eating disorder due to IF should not be neglected, as recent reports suggest a substantial correlation [152]. IF was shown to decrease testosterone and the free androgen index among males [153], which, in theory, could negatively affect metabolic health and libido.

Although further data are needed, especially from well-designed randomised controlled studies, it is obvious that different dietary fasting or caloric restriction regimens significantly impact the gut microbiota, both qualitatively and quantitatively. This should be seen in conjunction with other beneficial effects regarding health span parameters, mainly reported during CR and less during IF regimens.

Since more and more CR programmes are developed, further studies are needed to identify and differentiate between the long-lasting microbiota changes that could be used for a targeted and tailored dietary intervention. Also, future research should assess the different molecules produced by the gut microbiota as the result of specific types of CR programs or may investigate the combined effects of physical exercise and caloric restriction or of fasting and caloric restriction.

We posit that dietary restriction programmes could become important non-pharmacological interventions in the treatment of various diseases.

## Figures and Tables

**Table 1:** Preclinical studies regarding the potential effects of dietary intervention on the gut microbiota.

Animal Model	Intervention	Biospecimen	Microbiota Variations Assessment Method	Main Outcome on Microbiota	References
C57BL/6J mice, 6-week-old	12 weeks Control: food and water ad libitum(n = 8) HF(n = 8) CR (n = 8)	cecum	whole-genome, shotgun metagenomic sequencing	↑ Firmicutes, ↑ Actinobacteria, ↑ Firmicutes: Bacteroidetes ratio (in HF and CR) ↓ Bacteroidetes; ↓ *Parabacteroides* (in HF and CR) ↑ *Bifidobacteriaceae*,↑ *Lactobacillus johnsonii*, ↑ *Bifidobacterium pseudolongum*, ↑ *Faecalibaculum* (in CR)	[56]
Male C57BL/6 mice, 6-week-old	(a) Control (b) mice starved for 24 h (c) mice starved for 24 h and then fed ad libitum for 24 h	faeces	16S rRNA gene sequencing	In (b) and (c): ↑ *Akkermansia*, ↑ *Parabacteroides* ↑ *Muribaculum*, ↑ *Muribaculaceae* ↑ *Eubacterium coprostanoligenes* *↓ Lactobacillus*, *↓ Bifidobacterium*	[57]
Male Fisher 344 x Brown Norway hybrid F1 rats	Control: food and water ad libitum (n = 10) TRF Keto (n = 11) TRF Control (n = 12)	feces	16S rRNA gene sequencing	↓ Actinobacteria, ↓ Patescibacteria in TRF Keto and TRF Control (young and old) ↑ Verrucomicrobia in TRF Keto and TRF Control (young and old)	[58]
Asian toads	Hibernating Asian toads from 2 cities (TJ, n = 22; XZ, n = 23) [59]	small and/or large gut	16S rRNA gene amplicon sequencing	↑ Proteobacteria in the small intestine ↑ Bacteroidetes and Firmicutes in the large intestine	[60]
Rainbow trout	5 weeks Control: food and water ad libitum (n = 32) CR for 3 weeks, followed by 2 weeks of feeding (n = 32) IF for 3 weeks followed by 2 weeks of feeding (n = 32)	proximal intestine	16S rRNA gene sequencing (V3 region)	In CR and IF groups: ↑ *Helicobacter*, ↑ Bacteroidetes, ↑ Firmicutes ↓ Actinobacteria	[59]
C57BL/6 male mice, 7-week-old	11 weeks n = 6–7/group; (1) control (2) CR group (30% calorie restriction) (3) IF: 2-day fasting + 5-day ad libitum (4) IF^Ctrl^: 2-day fasting + 5-day feeding, with normal amounts of food	faeces	16S rRNA gene V3-V4 region	*Lactobacillus murinus* was the predominant bacterium in the CR, IF, and IF^Ctrl^ groups but barely present after fasting	[61]
Female Balb/c mice with allergies 6–8 weeks old	56 days Control: food and water ad libitum (n = 10) 16:8 IF (n = 10) 24: 24 IF (n = 10)	faeces	16s rRNA sequencing	24:24 group: ↑ *Alistipes*, ↑ *Eubacterium*, ↑ *Rikenellaceae-RC9-gut-group*, ↑ *Alloprevotella* 16:8 group: ↑ *Lachnospiraceae*-UCG-006 ↓ Firmicutes in both IF groups	[62]
NASH male C57BL/6 mice, 6-week-old	16 weeks WDAL: western diet ad libitum (n = 10) WDTRF: western diet time-restricted feeding (16:8) (n = 10) NDAL: normal chow diet ad libitum (n = 10) NDTRF: normal chow diet time-restricted feeding (16:8) (n= 10)	faeces	16S rRNA gene sequencing	WD treated mice (WDAL and WDTRF): ↓ *Bacteroidota*, *↓* Proteobacteria *↓ Cyanobacteria* ↑ Verrucomicrobia in WDTRF vs WDAL NDTRF group: ↑ *Lactobacillus* ↑ *Muribaculaceae* ↑ *Dubosiella*, ↑ *Clostridia* ↑ *Faecalibacterium*, *↓ Helicobacter*, *↓ Mucispirillum*	[63]
C57BL/6 mice, 4-week-old	M-IF female mice (n = 10), 12 weeks Control ad libitum (n = 10) O-IF ND: offspring from M-IF feed with a normal diet (n = 10) O-AL ND: offspring from ad libitum feed with a normal diet (n = 10)	ileum	16S rRNA gene sequencing	↓ *Lactobacillus intestinalis* in O-IF ND and O-AL ND	[64]
C57BL/6 mice with induced colitis	SIF, 2 weeks (n = 8) LIF, 20 weeks (n = 8) IF = fed every other day (n = 8) SAL (n = 8) LAL (n = 8)	feces	16S rRNA sequencing	SIF vs. SAL: ↓ Firmicutes, ↑ Bacteroidetes ↓ *Verrucomicrobia* in both SIF and LIF At family level (SIF vs. SAL): ↑ *Muribaculaceae*,↑ *Akkermansiaceae*, ↓ *Lachnospiraceae*,↓ *Ruminococcaceae* At genus level (SIF vs. SAL): ↑ *Bacteroides*, ↑ *Muribaculum*, ↑ *Akkermansia*, ↓ *Ruminiclostridium* LIF vs. LAD: ↓ *Akkermansiaceae*, ↑ *Lactobacillaceae*	[65]
Sprague–Dawley rats, 6–8 weeks old	T2DM group: fed ad libitum with HFD (n = 5) T2DM + CR (30% calories of the HFD) (n = 5) Control: food and water ad libitum (n = 5)	feces	16S rRNA gene sequencing	In T2DM + CR vs. T2DM: ↑ *Alistipes*, *↑Allobacum* ↑ *Lachnospiraceae*_NK4A136_group, ↑ *Ruminococcaceae_9*, ↓ *Bacteroides*, *↓ Lachnoclostridium*, *↓ Bifidobacterium*	[66]
Male C57BL/6 mice -intestinal ischemia/reperfusion model 6–8 weeks old	Preoperative fasting for 6, 12 or 24 h Control: food and water ad libitum (n= not mentioned)	faeces	16S rDNA sequencing, metabolomic analysis	Fasting group: ↓ *Helicobacter* ↑ *Ruminococcaceae-UCG-014*, ↑ *Akkermansia*, ↑ *Parabacteroides*, ↑ *Desulfovibrio*,↑ *Oceanisphaera*, ↑ *Psychrobacter*	[67]
Male Wistar rats, 12-month-old, high-fat diet-induced obesity	5 weeks IF(18/6F) (n = 7); Control: food and water ad libitum (n = 7)	cecum	Genomic DNA isolation	↓ Firmicutes: Bacteroidetes ratio, ↓ *Bacillus velezensis*, ↑ *Lachnospiraceae*, ↑ *Lactobacillaceae* (in IF group)	[68]
Male C57BL/6J mice 6-week-old	HF (60% fat) (n = 6) IF- fed every other day, 12 weeks (n = 6), Control: food and water ad libitum (n = 6)	ileum	16S rRNA gene V4 amplicon	↑ *Lactobacillus* in IF ↑ *Akkermansia muciniphila*	[69]
Male Sprague-Dawley rats (7–8 weeks) monocrotaline model of pulmonary arterial hypertension	24 days Control (n = 10) Monocrotaline-ad libitum feeding (n = 10) Monocrotaline-IF (every other day feeding) (n = 10)	faeces	16S ribosomal RNA gene V4-amplicon	in monocrotaline-IF: ↑ *Lactobacillus*	[70]
Male Wistar rats, 20 weeks old	4 weeks SC (n = 10) TC (n = 10) SIF (n = 10) TIF (n = 10)	feces	Incubation of colonies	↓ *Bifidobacterium*, ↓ *Lactobacillus*, ↓ *Enterococcus* (in TIF group) ↑ *Bifidobacterium*, ↑ *Enterococcus* (in SIF group)	[71]
C57BL/6N female mice, 8–10-week-old	SPF: female mice in specific pathogen-free conditions, 48 fasting or not (n = 5) GF: Germfree mice, 24 h fasting or not (n = 5) + oral infections *with S. Typhimurium* in SPF or GF mice	cecum	16S rRNA gene sequencing	↑ *Akkermansia*, ↓ *Bacilli*, ↓ *Erysipelotrichia* in the fasted mice	[72]

HF—high-fat diet; CR—caloric restriction; IF—intermittent fasting; (16:8)—16 h fasting/8 h feeding; (24:24)—24 h fasting/24 h feeding; WDAL—western diet ad libitum; WDTRF—western diet time-restricted feeding; NASH—notably alleviated obesity and nonalcoholic steatohepatitis; NDAL—normal chow diet ad libitum; NDTRF—normal chow diet time-restricted feeding; TRF—time-restricted feeding; M-IF—mother submitted to intermittent fasting; M-AL—mother fed ad libitum; O-IF ND—offspring from M-IF; normal diet; O-AL ND—offspring from M-AL; fed with a normal diet; SPF—specific pathogen-free; SIF—short-term intermittent fasting; LIF—long-term intermittent fasting; SAL—short-term ad libitum; LAL—long-term ad libitum; TJ—Tianjin (city); XZ—Xuzhou (city); T2DM—type 2 diabetes mellitus; STZ—streptozocin; SC—sedentary rats with access to feed ad libitum; TC—trained rats; SIF—sedentary rats submitted to intermittent fasting; TIF—trained rats were submitted to intermittent fasting.

**Table 2:** Main changes in the microbiota composition observed in the animal models.

	Healthy Mice [56]	Healthy Mice [57]	Healthy Mice [64]	Mice with Induced-Colitis (Short-Term IF) [65]	Mice with Induced- Colitis (Long-Term IF) [65]	Mice Ischemia/Reperfusion Model [67]	NASH Mice [63]	Mice Infected with *S. Typhimurium* [72]	Mice with Allergy (16:8 IF) [62]	Mice with Allergy (24:24 IF) [62]	Asian Toads [60]	Rainbow Trouts [59]	Healthy Rats [58]	Rats with HTA [70]	Trained Rats [71]	Obese Rats [68]	Obese Rats [69]	T2DM Model Rats [66]
Actinobacteria	▲											▼	▼					
*Akkermansia*		▲		▲	▼	▲		▲									▲	
*Alistipes*										▲								▲
*Allobaculum*													▼					▲
*Alloprevotella*										▲								
*Bacilli*								▼						▲		▼		
*Bacteroides*	▼			▲														▼
Bacteroidetes	▼			▲							▲	▲						
*Bifidobacterium*		▼													▼			▼
*Butyricicoccus*														▼				
*Clostridium* spp.							▲							▲				
*Dubosiella*							▲											
*Enterococcus*														▲	▼			
*Eubacterium* spp.		▲								▲			▼					
*Faecalibacterium*							▲											
Firmicutes	▲			▼		▼			▼	▼	▲	▲						
*Helicobacter*						▼	▼					▲						
*Lachnospiraceae*				▼					▲							▲		▲
*Lactobacillus*		▼	▼		▲		▲							▲	▼	▲	▲	
*Ligactobacillus*																▲		
*Muribaculaceae*		▲		▲			▲											
*Parabacteroides*	▼	▲				▲												▼
*Prevotella*														▼				
Proteobacteria						▲					▲							
*Rhudospirillacea*														▼				
*Rikenellaceae*										▲								
*Ruminococcaceae*				▼		▲												▲
Verrucomicrobia		▲		▼	▼								▲					
*Lachnoclostridium*																		▼

▲ Increased ▼ Decreased.

**Table 3:** Clinical studies regarding the potential effects of dietary intervention on the gut microbiota.

Design	Study Population(s)	Dietary Intervention	Main Outcome on Microbiota	References
A randomised, controlled, single-blinded study	151 healthy volunteers	5 days, Buchinger fasting program (n = 20) Fasting mimetic (n = 100) Control (n = 31)	Buchinger fasting: ↑ Proteobacteria ↓ Firmicutes/Bacteroides ratio Sirtfood intervention: ↑ Actinobacteria	[88]
A randomised, controlled, single-blinded trial	51 healthy volunteers	5 days, Buchinger fasting program (n = 20) Control (n = 31)	↓ Firmicutes/Bacteroides ratio ↑ *Christensenellaceae* species	[89]
Intervention pre-and post-design	16 healthy volunteers	water-only fast (n = 6) juice fast (n = 10) 7 days	The water-only fasting: ↓ Fusobacterium ↑ homogenous gut microbiota	[90]
Longitudinal physiologic data in 2 cohorts sampled in 2 different years	67 young and middle age healthy volunteers	Ramadan IF young (n = 30) Ramadan IF middle age (n = 27) Control (n = 10) 30 days	↑ alpha diversity only in the younger group ↑ *Lachnospiraceae*, ↑ *Ruminococcaceae* ↓ Bacteroidales (Prevotellaceae)	[7]
Pilot trial	15 healthy volunteers	10 days, Buchinger fasting programme, and subsequent 3-month refeeding	↓ Firmicutes (*Lachnospiraceae* and *Ruminococcaceae*) ↑ Bacteroides, Proteobacteria (*Escherichia coli*, *Bilophila wadsworthia*)	[91]
Pilot trial	9 volunteers	Ramadan IF, 29 days	↑ *Faecalibacterium prausnitzii*, *Roseburia*, *Eubacterium*, and *Akkermansia*, *Bacteroides* ↑ *Butyricicoccus pullicaecorum*	[92]
Pilot trial	9 volunteers	Ramadan IF, 29 days	↑ *A. muciniphila* and *Bacteroides fragilis*	[23]
Cohort trial	34 volunteers (16 Chineseand 18 Pakistaniadults)	Ramadan IF, 29 days	↓ *Coprococcus*, *Clostridium_XlV* spp., *Lachnospiracea* (both groups) ↑ *Dorea*, *Klebsiella*, *Faecalibacterium* (Chinese group) ↑ *Sutterella*, *Parabacteroides*, *Alistipes*, *Bacteroides* (Pakistani group)	[93]
Cross-sectional study	30 healthy subjects	Ramadan IF, 29 days	↑ *Bacteroides* (both sexes) ↑ Firmicutes (only women) ↑ serum levels of butyrate	[94]
Cohort trial	34 volunteers (16 Chinese; 18 Pakistanis)	Ramadan IF, 29 days	↑ L-histidine, cordycepin, lycofawcine (Chinese group) ↑ brucine (Pakistani group)	[95]
Cohort trial	30 healthy men	TRF (n = 15) non-TRF (n = 15).	↑ Bacteroidetes phylum (*Prevotella_9*, *Faecalibacterium*, *Dialister*) in TRF group	[96]
Pilot trial	13 obese patients	7 days Buchinger fasting program and laxative followed by 6 week probiotic formula	↑ Faecalibacterium prausnitzii, ↑ Akkermanis, ↑Bifidobacteria	[97]
A randomised controlled trial	35 hypertensive patients with metabolic syndrome	5-days Buchinger fasting program, followed by a Mediteran-like diet for 3 months	↓ *Bifidobacterium*, *Coprococcus comes*, *Roseburia* ↑ *Faecalibacterium prausnitzii*, *Bacteroides*, *Anaerotruncus* ↑ propionate production capacity and mucin degradation gene modules	[98]
A randomised controlled trial	39 patients with metabolic syndrome	IF group (n = 21) Control group (n = 18) 8 weeks	↑ SCFA levels ↑ *Ruminococcaceae*, *Roseburia*, and *Clostridium* ↓ lipopolysaccharides	[99]
Intervention pre-and post-design	71 underweight, normal, or obese volunteers	Women (n = 40) Men (n = 31) TRF regimen (16:8) for 16 days.	↑ alpha diversity ↑ *Bifidobacteria*, *Lactobacillus* (all groups) ↓ Firmicutes, (obese women) ↑ Proteobacteria, Bacteroidetes, Actinobacteria (all women groups) ↑ Firmicutes (normal/underweight female groups) ↓ Firmicutes, Actinobacteria (male normal group) ↑ Proteobacteria, Bacteroidetes (male normal group) ↑ Firmicutes, Bacteroidetes, and Actinobacteria (underweight group) ↓ Proteobacteria (underweight group)	[100]
Prospective cohort study	10 obese postmenopausal women	VLCD (800 kcal/day) for 46 days	↓ *Faecalibacterium prausnitzii*, *Roseburia* genus ↑ *Christensenellaceae*	[101]
Cohort trial	49 overweight and obese adults	6-week caloric restriction and 6-week weight stabilisation diet	↓ *Akkermansia muciniphila* (Akk HI group)	[102]
Pilot trial	12 obese type 2 diabetics	VLCD, 6 weeks 15 week follow up	↑ alpha diversity ↓ *Collinsella* genus, *Roseburia*, *Lachnospiraceae* spp. changes observed had reverted until the end of the follow-up	[103]
Randomised trial	16 obese patients	6-week VLCD+ 12 months follow-up period	↓ *Bacteroides* spp., *Lactobacillus*, but the change was transient.	[104]
Cross-sectional study	obese/overweight adolescent patients (n = 13) Lean (n = 8)	Long-term CR (1700 kcal/day), 12 months	↓ Actinobacteria, ↓ Firmicutes:Bacteroidetes ratio ↑ *Bacteroides*, *Roseburia*, *Faecalibacterium* and *Clostridium XIVa*	[105]
Multicentre trial	211 obese/pre-diabetic patients	CR (800–1200 kcal/day), 8 weeks	↑ *Akkermansia* and *Christensenellaceae* R-7 group ↓ *Blautia*, *Bifidobacterium* spp.	[106]

VLCD—very low-calorie diet; CR—caloric restriction; IF—intermittent fasting; TRF—time restricted fasting.

**Table 4:** Summary of human studies investigating comparability between various dietary interventions and effects on gut microbiota.

Design	Population	Dietary Regimens	Main Findings	References
Randomised trial	59 overweight or obese adults	CR (n = 25) IF (n = 34) 3 months	↓ *Subdoligranulum* and *Collinsella* ↑ *Parabacteroides*, *Alistipes,* and *Bacteroides* (CR and IF) ↑ *Akkermansia* (only in IF)	[116]
Real-life study	49 obese patients	TRF (n = 25) CR(n = 24) 12 weeks	No differences in α and β diversity. ↑ *Lachnospiraceae*, *Parasutterella*, and *Romboutsia (TRF group)*	[117]
Controlled parallel design trial	61 obese patients	VLCKD (n = 18), 2 months BS (n = 22) MetDiet (n = 21) 6 months	↑ *Clostiridiaceae* (MetDiet, VLCKD) ↑ *Parabacteroides* and *Alistipes* (VLCKD, BS) ↓ *Lactobacillus* (MetDiet, BS, VLCKD)	[118]
Randomised trial	62 overweight or obese patients	CCR (n = 27) IF (n = 35) 12 months	Weight loss was correlated with *Ruminococcaeae* NK4A214 and *Coprococcus* 3 baseline abundance. Bacteroides and Lachnospiraceae levels were considered disadvantageous for weight loss. *Faecalibacterium* and *Blautia* baseline abundances were associated with a decrease in triglicerides	[119]
A parallel-arm randomised controlled study	147 overweight or obese adults	IF 5:2 (n = 47) CCR (n = 46) Control (n = 51) 50 weeks	↑ Lactobacillus (IF) Associations between HOMA-IR and Akkermansiaceae, Christensenellaceae, and Tanerellaceae	[120]

BS—bariatric surgery; CCR—continuous caloric restriction; CR—caloric restriction; IF—intermitent fasting; MetDiet—hypocaloric Mediterranean diet; TRF—time restriction fasting; VLCKD—very-low-calorie ketogenic diet.

**Table 5:** Main changes in the microbiota composition observed in the human population.

	Healthy Chinese; Ramadan [86]	Healthy Pakistani; Ramadan [86]	Healthy (Both Groups); Ramadan [86]	Healthy; Buchinger [83]	Healthy Volunteers; IF(16:8) [90]	Healthy Volunteers; Buchinger [80]	Healthy; Ramadan [88]	Healthy Volunteers; Ramadan [23]	Healthy Volunteers; Ramadan [85]	Healthy Volunteers; Ramadan [7]	Obese Women; VLCD [95]	Obese Patients; TRF [106]	Obese Patients; VLCD [102]	Obese Patients; VLCKD [107]	Metabolic Syndrome; IF(5:2) [94]	Obese/Pre-Diabetic; CR [104]	Obese Patients; IF(16:8) [99]	HTA/metab. syn; Buchinger [93]	Obese Patiemts; Buchinger [92]	Obese Adolescents; CR [97]	Overweight Patients; CR [105]	Obese Patients; IF(5:2) [109]
Actinobacteria													▼				▲			▼		
*Actinomyces*						▲																
*Akkermansia*								▲	▲							▲			▲		▲	
*Alistipes*		▲		▲										▲							▲	
Alpha diversity									▲	▲			▲						▲		▲	
*Anaerotruncus*				▲																		
*Bacteroides*	▼	▲		▲	▲		▲	▲									▲	▲			▲	
*Bifidobacterium*																▼	▲	▼	▲			
*Blautia*		▼														▼						
*Butyricoccus*									▲						▲							
*Christensellaceae* spp.						▲					▲			▲		▲						
*Clostridium XIV* spp.			▼											▲						▲		
*Collinsella*													▼								▼	
*Coprococcus*		▼	▼	▼																		
*Dialister*		▼							▲													
*Dorea*	▲													▲								
*Eggerthella*													▲									
*Eubacterium*		▼							▲	▲												
*Faecalibacterium*	▲			▼	▲				▲	▲	▼									▲		
*Lachnospiraceae* spp.			▼							▲		▲	▼	▼								
*Lactobacillus*																			▲			▲
*Oscillibacter*				▼																		
*Parabacteroides*		▲												▲							▲	
*Prevotella*					▲					▼												
*Pseudoflavonifractor*													▲									
*Romboutsia*		▼								▼		▲					▲					
*Roseburia*				▼					▲		▼		▼	▼	▲					▲		
*Ruminococcaceae* spp.															▲							
*Streptococcus*		▼											▼									
*Subdoligranulum*										▲											▼	

▲ Increased ▼ Decrease.

## Data Availability

Data are contained within the article.
